# Characterization of blue light irradiation effects on pathogenic and nonpathogenic *Escherichia coli*

**DOI:** 10.1002/mbo3.466

**Published:** 2017-03-22

**Authors:** Courtney M. Abana, John R. Brannon, Rebecca A. Ebbott, Taryn L. Dunigan, Kirsten R. Guckes, Hubaida Fuseini, Jennifer Powers, Bridget R. Rogers, Maria Hadjifrangiskou

**Affiliations:** ^1^ Department of Chemical & Biomolecular Engineering Vanderbilt University Nashville TN USA; ^2^ Department of Pathology Microbiology and Immunology Vanderbilt University Medical Center Nashville TN USA; ^3^ Department of Biological Sciences Vanderbilt University Nashville TN USA; ^4^ Department of Biomedical Engineering Vanderbilt University Nashville TN USA; ^5^ Vanderbilt Dermatology Vanderbilt University Medical Center Nashville TN USA; ^6^ Department of Urologic Surgery Vanderbilt University Medical Center Nashville TN USA; ^7^ Present address: Duke Dermatology Duke University School of Medicine Durham NC USA

**Keywords:** *E. coli*, membranes, pathogenesis, persisters

## Abstract

Blue light irradiation (BLI) is an FDA‐approved method for treating certain types of infections, like acne, and is becoming increasingly attractive as an antimicrobial strategy as the prevalence of antibiotic‐resistant “superbugs” rises. However, no study has delineated the effectiveness of BLI throughout different bacterial growth phases, especially in more BLI‐tolerant organisms such as *Escherichia coli*. While the vast majority of *E. coli* strains are nonpathogenic, several *E. coli* pathotypes exist that cause infection within and outside the gastrointestinal tract. Here, we compared the response of *E. coli* strains from five phylogenetic groups to BLI with a 455 nm wavelength (BLI_455_), using colony‐forming unit and ATP measurement assays. Our results revealed that BLI_455_ is not bactericidal, but can retard *E. coli* growth in a manner that is dependent on culture age and strain background. This observation is critical, given that bacteria on and within mammalian hosts are found in different phases of growth.

## 1 Introduction

The steady increase in antibiotic resistance rates among bacteria has sparked a major research effort to identify new antibacterial and antivirulence therapies (Bebell & Muiru, 2014; Brooks & Brooks, 2014; Roca et al., 2015; Thabit, Crandon, & Nicolau, 2014; Uchil, Kohli, Katekhaye, & Swami, 2014). Phototherapy is among these alternative approaches and is used routinely to treat skin infections, like acne (Dai et al., 2012; Dong et al., 2016; Liu et al., 2016; Maisch et al., 2014; Opel et al., 2015; Pei, Inamadar, Adya, & Tsoukas, 2015; Piccolo et al., 2014). Phototherapy employs visible light in the 400–700 nm wavelength range to activate either exogenous (as in photodynamic therapy‐PDT) or endogenous photosensitizers (PSs) (Lambrechts, Demidova, Aalders, Hasan, & Hamblin, 2005; Maclean, Macgregor, Anderson, & Woolsey, 2008, 2009; Nussbaum, Lilge, & Mazzulli, 2002; Orenstein et al., 1997). Endogenous bacterial PSs, such as porphyrins and flavins, absorb light in the 400–500 nm blue light wavelength range and are suggested to respond to blue light irradiation (BLI) (Cieplik et al., 2014; Dai et al., 2012; Lubart, Lipovski, Nitzan, & Friedmann, 2011).

Previous reports suggested that irradiation with visible light without the use of exogenous PSs kills species like *Acinetobacter baumannii*,* Aggregatibacter actinomycetemcomitans*,* Enterococcus faecalis*,* Escherichia coli*,* Porphyromonas gingivalis*,* Pseudomonas aeruginosa*,* Staphylococcus aureus*, and *Streptococcus pyogenes* by as much as 90% after exposure (Bumah, Masson‐Meyers, Cashin, & Enwemeka, 2013; Buonanno et al., 2013; Cieplik et al., 2014; Donnelly et al., 2009; Enwemeka, Williams, Enwemeka, Hollosi, & Yens, 2009; Gad, Zahra, Francis, Hasan, & Hamblin, 2004; Guffey & Wilborn, 2006a,b; Hamblin et al., 2005; Lipovsky, Nitzan, Gedanken, & Lubart, 2010; Lubart et al., 2011; Maisch et al., 2014; Nitzan, Malik, Kauffman, & Ehrenberg, 1997; Perni et al., 2009; Soukos et al., 2005; Zolfaghari et al., 2009). These studies included wavelengths ranging from 207 to 880 nm and energy doses ranging from 1 to 420 J/cm^2^. A lower wavelength corresponds to a higher energy per particle of light (photon). The energy dose is defined by the total energy delivered per irradiated area. At a constant energy dose, the number of photons increases as the wavelength increases. *A. actinomycetemcomitans*,* E. coli*,* P. gingivalis*,* Porphyromonas intermedia*,* Porphyromonas nigrescens*,* Prevotella melaninogenica*,* S. aureus*, and *Streptococcus constellatus* were reported to be killed using light at 455 nm (+/‐ 5 nm) without the use of exogenous PSs and 4–150 J/cm^2^ (Cieplik et al., 2014; Lipovsky et al., 2010; Soukos et al., 2005). However, in all studies, bacterial viability was measured only as a function of colony‐forming units (CFUs), therefore, not accounting for the potential formation of viable but nonculturable subpopulations.

Among the potential endogenous PSs harbored by bacteria, are porphyrin‐containing cytochromes, ubiquinones, and flavin‐containing enzymes, such as flavin adenine nucleotide (FAD). Cytochromes and flavin‐containing enzymes are integral components of the bacterial electron transport chain (ETC). For instance, the reduced form of FAD, FADH_2_, is utilized as an electron donor through an oxidative reaction resulting in FAD (Fig. S1a and (Cook, Greening, Hards, & Berney, 2014; Richter & Ludwig, 2009; Sazanov, 2014)). Cytochromes and ubiquinones, serve as both electron acceptors and mobile electron carriers in the ETC (Fig. S1a and (Aussel et al., 2014; Cook et al., 2014; McNeil & Fineran, 2013; Richter & Ludwig, 2009; Sazanov, 2014)). The aromatic rings within electron donors and carriers absorb photons from light, which makes them ideal endogenous PSs (Herzfeld, 1940). Flavins have absorption peaks at 380 and 450 nm, porphyrins around 400 nm, while ubiquinones have various peaks in the 230–500 nm range (Chandra et al., 2000; Land, Simic, & Swallow, 1971; Yagi, Ozawa, & Harada, 1959; Zenichowski, Gothe, & Saalfrank, 2007).

Previous studies indicated that light promotes PSs to an excited state, after which electrons (or energy) are transferred to molecules such as molecular oxygen, forming reactive oxygen species (ROS) (Fig. S1b and (Feuerstein, Ginsburg, Dayan, Veler, & Weiss, 2005; Liang et al., 2013; Lubart et al., 2011; Malik, Hanania, & Nitzan, 1990; Marugán, van Grieken, Pablos, & Sordo, 2010)). Increased ROS levels can cause damage to cellular proteins, lipids, and DNA (Farr & Kogoma, 1991; Feuerstein, Moreinos, & Steinberg, 2006; Liang et al., 2013; Lipovsky, Nitzan, Friedmann, & Lubart, 2009; Lipovsky et al., 2010).

To date, most studies focused on the antimicrobial effects of BLI were performed using bacteria in the exponential growth phase (Cieplik et al., 2014; Feuerstein et al., 2005; Guffey & Wilborn, 2006a,b; Lambrechts et al., 2005; Lipovsky et al., 2009, 2010; Nitzan, Gutterman, Malik, & Ehrenberg, 1992; Orenstein et al., 1997; Yang, Inokuchi, & Adler, 1995; Yin et al., 2015). Many elegant studies have now demonstrated that in the majority of infections, the inoculating bacteria are at extremely low concentrations (Dupont, Levine, & Hornick, 1989; Gonzalez, Lane, Wagner, & Weening, 2015; Griffin & Tauxe, 1991; Handley, Dube, & Revell, 2004; Kotloff, Losonsky, Nataro, & Wasserman, 1995; Lorange, Race, & Sebbane, 2005; Tacket, Binion, & Bostwick, 1992) and that bacteria are not always exponentially growing (Helaine & Holden, 2013). In many respects, stationary phase bacteria differ from their exponential phase counterparts (Anderl, Zahller, Roe, & Stewart, 2003; Cabeen, 2014; Kolter, Siegele, & Tormo, 1993; Mangan et al., 2006; Mouslim & Hughes, 2014; Navarro Llorens, Tormo, & Martínez‐García, 2010; Roop et al., 2003; Wang et al., 2014). For instance*, E. coli, P. aeruginosa*, and *S. aureus* cultures have a low, stable subpopulation of “persister” cells during lag and early‐exponential phases, and this subpopulation increases during mid‐exponential and stationary phases (Keren, Kaldalu, Spoering, Wang, & Lewis, 2004). Persister cells are phenotypically distinct from their genetically identical, active sister cells (Bigger, 1944; Keren, Shah, Spoering, Kaldalu, & Lewis, 2004; Keren, Kaldalu, et al., 2004) and their dormant state allows them to tolerate the presence of antibiotics (Bigger, 1944; Keren, Kaldalu, et al., 2004; Keren, Shah, et al., 2004). In addition to persister cells, there is an increasing body of literature demonstrating extensive population heterogeneity in bacterial communities during infection, which also changes as the culture ages (Balaban, Gerdes, Lewis, & McKinney, 2013; Bush et al., 2011; Darby, Venugopal, Ehrt, & Nathan, 2011; Floyd et al., 2015; Hisert et al., 2004; Lechner, Lewis, & Bertram, 2012; Moy et al., 2006; Smith, Nathan, & Peavy, 2005; Tian, Bryk, Itoh, Suematsu, & Nathan, 2005). Considering the dynamic flux in bacterial subpopulations during growth, successful translation of BLI into potential antibacterial applications requires an assessment of the bacterial response to BLI during different growth phases.

In this study, we evaluated the effects of BLI at a 455 nm wavelength (BLI_455_) without the use of exogenous PSs on the growth and viability of different *E. coli* strains from different phylogenetic groups. We performed our analyses during exponential, transition, and early stationary phases of growth to encompass responses to BLI that differ based on bacterial growth phase. We selected BLI_455_ for investigation, as this wavelength is within the range of FDA‐approved phototherapy devices, including “bili‐blankets” used to treat jaundiced neonates (Morris et al., 2013; Sherbiny, Youssef, Sherbini, El‐Behedy, & Sherief, 2015). Our results indicated that although BLI_455_ reduced the number of growing *E. coli* cells to varying degrees in a strain‐ and growth phase‐dependent manner, BLI did not significantly impact the viability of any strain. Rather, BLI treatment induced a viable but nonculturable state, which may influence chronic infection states in the hospital setting.

## 2 Experimental Procedures

### 2.1 Strains and constructs

The following *E. coli* strains were used: phylogenetic group A strains DH5α [laboratory‐adapted (Laboratories, 1986)] and MG1655 [K‐12 (Bachmann, 1996)]; group B1 strains E343 and E402 [nonpathogenic isolates (Rúgeles, Bai, Martínez, Vanegas, & Gómez‐Duarte, 2010)]; group B2 UPEC strains UTI89 (Mulvey et al., 1998) and EC958 (Totsika et al., 2011); group D enterotoxigenic (ETEC) strain E9034A (Levine et al., 1984); and group E enterohemorrhagic (EHEC) O157:H7 strain Sakai (Hayashi et al., 2001).

### 2.2 Culture conditions

Bacterial cultures were seeded in LB and incubated at 37°C while shaking overnight (Bertani, 1951). Aliquots of overnight cultures were normalized to an optical density at 600 nm (OD_600_) of 0.05 into LB subculture in 125 ml flasks. Subcultures were incubated at 37°C and shaken at 200 RPM for all analyses described.

### 2.3 Growth curves

Bacteria were inoculated as described in the section above. A 120‐μl aliquot was removed from each culture hourly. Of each aliquot, 100 μl were diluted 1:10 in fresh LB and the OD_600_ was recorded using a Thermo Scientific NanoDrop 2000 spectrophotometer. The remaining 20 μl were then serially diluted in LB for enumeration of colony‐forming units (CFUs). A multichannel pipette was used to spot eight different dilutions with five technical replicates per dilution. Incubation occurred overnight at room temperature (RT). Growth curve experiments were repeated at least three independent times, with a minimum of five technical replicates per biological repeat. Generation times were calculated using Equation (1), where *G* is generation time, *t* is the time interval, *b* is the CFU of bacteria at the end of the time interval, and *B* is the CFU of bacteria at the beginning of the time interval.

(1) $$G=\frac{t}{3.3\log(b/B)}$$

### 2.4 *In vitro* light delivery to *E. coli*

Cultures were set up as described above. Aliquots were obtained for irradiation and plating during exponential (*t* = 3 hr), transition (*t* = 5 or 6 hr depending on the strain), and stationary (t = 8 hr) growth phases. Aliquots were serially diluted as described above and 10 μl from one serial dilution (~10^1^ to 10^2^ cells) was spotted on solid LB agar and exposed to BLI_455_. BLI_455_ was carried out with a Thorlabs Mounted High‐Power 455 nm LED lamp and controlled by a high‐powered LED driver (Thorlabs DC2100). The light source was placed 10 mm ± 1 mm above the 10‐μl spots, to achieve a power flux output of ~520 mW/cm^2^. A total energy dose of 120 J/cm^2^ was delivered to each sample. Irradiated and nonirradiated controls were then incubated overnight at ambient temperature. CFUs were counted the following day. Experiments were performed with at least three biological replicates of three technical replicates.

### 2.5 Survival fraction determination

To determine the percent change in exposed versus unexposed CFUs, the CFUs postirradiation were enumerated and compared to the CFUs of corresponding, nonirradiated spots. The reductions were calculated using Equation (2).

(2) $$\text{Percent reduction in CFUs}\;\left(\%\right)=\left[1-\frac{\text{exposed CFUs}}{\text{unexposed CFUs}}\right]\times 100\%$$

### 2.6 Viability assay

Cell viability was determined using the CellTiter‐Glo Luminescent Cell Viability Assay kit (Promega). Two assays were performed: one to determine the overall differences in ATP of unexposed and exposed samples and the second to determine if differences in ATP amounts of unexposed and exposed samples were due to cell death (cell lysis) or inhibition of replication. For both assays, 50 μl of liquid culture was placed on a glass cover slip directly under the light source. The height between the light source and the sample was adjusted to deliver 120 J/cm^2^ for the increased irradiated area because of the increased volume and spread on the glass slide, compared to the spread on agar plates. After BLI_455_, the sample was transferred to a 96‐well plate and allowed to incubate for 30 min, allowing for at least one replication cycle to occur. After incubation in the first assay, triplicate samples of unexposed and exposed aliquots were diluted 10‐fold in LB and 50 μl of each diluted sample was transferred to a black, 96‐well plate (Costar). In the second assay, 200 μl of unexposed and exposed samples (previously diluted 10‐fold after the 30 min incubation) were collected in separate 1.5 ml plastic tubes for each strain. The plastic tubes were placed in a centrifuge for 2 min at 16,100*g* to pellet cells. After centrifugation, the supernatant was transferred to a new 1.5 ml plastic tube. The pellets were then resuspended in 200 μl of LB. Triplicate samples (50 μl each) of supernatant or resuspended pellet were added to individual wells in a black well, 96‐well plate (Costar) and quantified as follows. For both assays, an equal volume of CellTiter‐Glo substrate/buffer mix was added to each well and mixed thoroughly. After addition of the CellTiter‐Glo, the plates were allowed to shake orbitally for 2 min to stabilize the signal and then luminescence values of ATP were measured using a SpectraMax i3 (Molecular Devices). Luminescence was also determined for wells filled only with LB to subtract background luminescence due to the media. Experiments were performed with at least three biological replicates of three technical replicates.

### 2.7 Biofilm assay

Strains were grown exponentially in 3 ml LB and normalized to an OD_600_ of 1. Cultures were then diluted 200‐fold in fresh LB and used to seed biofilm plates. Biofilm assays in LB at RT were performed in 96‐well PVC plates as previously described (Pinkner et al., 2006) and quantitatively measured 24 hr post seeding, using crystal violet (O'Toole et al., 1999).

The effect of BLI on preformed colony biofilms was also evaluated. Overnight cultures were normalized to a starting OD_600_ of 0.05. Agar plates were spotted with 10 μl of bacterial inoculum. Plates were left to grow in the dark at RT for 3 days. After 3 days of growth, half of the plates were irradiated with BLI_455_ at 120 J/cm^2^. Plates were then placed in the dark at RT for two additional days. On day 5 post seeding, the diameters of unexposed and exposed biofilms were measured. This assay was repeated with three biological replicates of each strain.

### 2.8 Persister assay

Persister assays were performed using ofloxacin, as described in (Allison, Brynildsen, & Collins, 2011). For the control experiments, stationary phase cultures of WT UTI89 and a mutant with known reduced proton motive force production, UTI89Δ*visC* (Conlon et al., 2016; Floyd et al., 2016) were subjected to 5 μg/ml ofloxacin for 4 hr. Samples were withdrawn for CFU enumeration prior to ofloxacin exposure. Susceptibility assays were repeated three times. The average percent survival from the three independent experiments is reported. For analysis of the BLI‐treated bacteria, the method for “In vitro light delivery to *E. coli*” was followed prior to the start of this assay. One colony was chosen from all technical and biological replicates of unexposed and exposed plates. Glass test tubes with 2 ml of LB were inoculated with one colony each from both the unexposed and exposed plates. Samples were placed into a shaking incubator at 37°C for 3 hr. After the 3‐hr incubation, two 750‐μl samples were aliquoted from glass test tubes and were placed in plastic 1.5 ml tubes. One of the 750‐μl aliquots was treated with ofloxacin (5 μg/ml). Plastic tubes were placed back into the shaking incubator for an additional 2‐hr incubation. After the second incubation, samples were centrifuged at 18,000*g* RPM for 2 min and washed in LB to reduce the carry‐over effects of ofloxacin. Aliquots of samples were serially diluted and plated for CFU enumeration. The average percent survival is reported.

## 3 Results

### 3.1 Strain‐ and growth phase‐specific responses to BLI_455_ for *E. coli*

**Figure 1:**
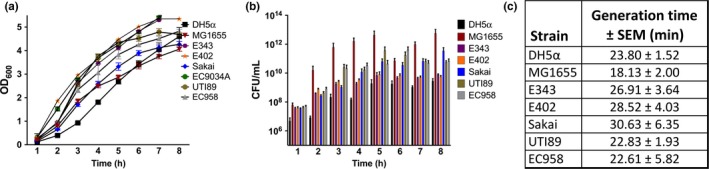
Growth rate heterogeneity exists between *E. coli* strains. Growth curves of *E. coli* strains ranging from nonpathogenic laboratory‐engineered strain DH5α; commensal strains MG1655, E343, and E402; enterohemorrhagic (EHEC) strain Sakai; and uropathogenic *E. coli* (UPEC) strains UTI89 (cystitis isolate) and EC958 (multi‐drug‐resistant isolate). Measuring the optical density alone can sometimes be misleading, depending on the surface factors of a particular strain; therefore, the growth curve is presented as (a) Optical density at 600 nm (OD_600_) versus time and (b) colony‐forming units per milliliter (CFU/ml) versus time for the seven strains. (c) To account for the varying growth rates, the generation time (plus the standard error mean‐SEM) was measured between hours 2 and 4 for each strain. MG1655 has the fastest growth rate (lowest generation time); however, looking at OD600 alone, MG1655 appears to grow slower than every other strain tested. Growth curve experiments were repeated at least three times independently. Error bars represent the standard error mean

Much of the previous work evaluating the efficacy of BLI in growth reduction of *E. coli* and other gram‐negative bacteria investigated only one strain from each bacterial species (Alves et al., 2015; Hanakova et al., 2014; Liang et al., 2013; Nussbaum et al., 2002; Popov et al., 2005; Tschowri, Busse, & Hengge, 2009; Tschowri, Lindenberg, & Hengge, 2012). While analyzing a single, model strain provides an initial basis for comparison, understanding how different strains within a species respond to BLI is essential to develop successful therapeutic approaches against them, especially given the strain heterogeneity within species like *E. coli* (Croxen & Finlay, 2010). *E. coli* strains belong to different phylogenetic groups, of which B2 and D harbor most pathogenic strains, while B1 mostly comprises nonpathogenic strains. We selected a range of strains from A, B1, B2, D, and E phylogenetic groups for our analyses. The different strains were first evaluated for growth rate differences to pinpoint exponential, transition, and stationary phase times for each one (Figure**** 1). These analyses revealed differences in the growth rates of these strains, with the nonpathogenic strain MG1655, a K‐12 derivative, exhibiting a generation time of ~18 min during exponential phase (Figure**** 1c). The uropathogenic *E. coli* (UPEC) isolates UTI89 and EC958, had nearly identical generation times (~22.6–22.8 min); while, the gastrointestinal *E. coli* strains exhibited slower generation times (Figure**** 1c).

**Figure 2:**
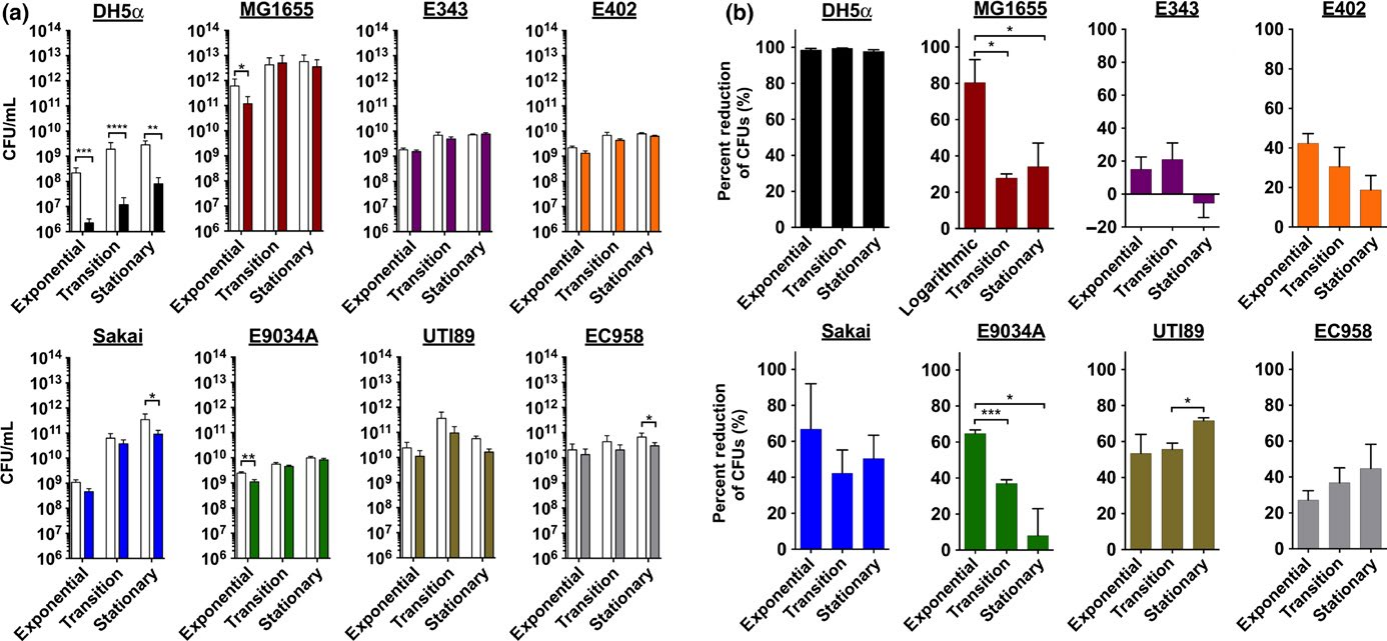
CFU reduction of different *E. coli* strains in response to blue light irradiation (BLI)_455_ at different growth phases. (a) Comparison of CFUs for unexposed (solid colored bars) and exposed (white bars) samples during exponential, transition, and stationary growth phases. All experiments were repeated a minimum of three times and analyzed via a Student *t* test. *, *p* < .05, **, *p *<* *.01; ***, *p *<* *.001; ****, *p *<* *.0001. (b) Comparison of percent reduction of CFUs with analyses of the changes over growth phases. The following strains are represented: phylogenetic group A strains DH5α (laboratory‐adapted) and MG1655 (K‐12); group B1 strains E343 and E402 (nonpathogenic isolates); group B2 UPEC strains UTI89 and EC958 (multi‐drug resistant); group D ETEC strain E9034A; and group E EHEC O157:H7 strain Sakai. Data represent the mean of three or more independent experiments. All experiments were repeated a minimum of three times and analyzed via One‐way anova. *, *p *<* *.05; ***, *p *<* *.001

We then tested each strain for susceptibility to BLI_455_ at their corresponding exponential, transition, and stationary growth phases, using the workflow depicted in Fig. S2. Figure**** 2 depicts the obtained results as a function of exposure (Figure**** 2a) and growth phase (Figure**** 2b). Of all strains tested, only DH5α displayed ~1.5–2.5 logs of decrease in CFUs following BLI, and this reduction was conserved during all phases of growth (Figure**** 2). During stationary and transition phase, MG1655 exhibited statistically insignificant changes as a result of BLI_455_; though, nearly a 1‐log decrease in the amount of CFUs from the unexposed to the exposed samples was observed for this strain during exponential phase. The commensal E343 and E402 and multi‐drug‐resistant UPEC strain EC958 exhibited modest, though statistically insignificant, susceptibility to the effects of BLI_455_ during all growth phases (Figure 2). The enterotoxigenic (ETEC) E9034A was more susceptible to BLI_455_ in exponential phase compared to transition and stationary phases where CFUs became minimally reduced (Figure 2). UPEC strains UTI89 and EC958, and EHEC strain Sakai were most susceptible in stationary phase (Figure 2). These data indicate that there are strain‐dependent responses to BLI_455_.

### 3.2 BLI_455_ and 120 J/cm^2^ is not bactericidal against *E. coli*

The minimal reduction in CFUs observed in our analyses could be the result of bacterial cell lysis and/or altered bacterial growth, such as the formation of persister cells. Following a modified method of Allison et al., we assessed the effects of BLI on the formation of persister cells. We first analyzed the amount of persister cells formed by UPEC strain UTI89 (Fig. S3A). In contrast to numbers obtained for nonpathogenic *E. coli* (Allison et al., 2011), UTI89 forms close to 4% persister cells upon a 4 hr exposure to ofloxacin. However, this survival was minimally affected upon BLI treatment (Fig. S3B), indicating that under the conditions tested, the reduction in CFUs observed is not due to the formation of persister cells.

We next determined whether BLI treatment leads to another form of growth arrest, or whether it leads to bacterial cell lysis. In the case of bacterial cell lysis, membranes become compromised, resulting in release of ATP to the extracellular milieu. On the other hand, altered bacterial growth could result from a perturbation in proton flux across the inner membrane, which would lead to an overall reduction in ATP production via the ETC. We used an ATP quantitation assay to determine how BLI_455_ impacts total, intracellular and extracellular ATP levels (Fig. S4 and S5). To enable accurate ATP measurements, the sample volume irradiated for the ATP assays was 50 μl, compared to 10 μl used to quantify the ability of BLI_455_ to reduce bacterial growth. The percent reduction in CFUs in response to BLI_455_ was determined for both volumes, using a representative set of strains (Fig. S6**)**.

**Figure 3:**
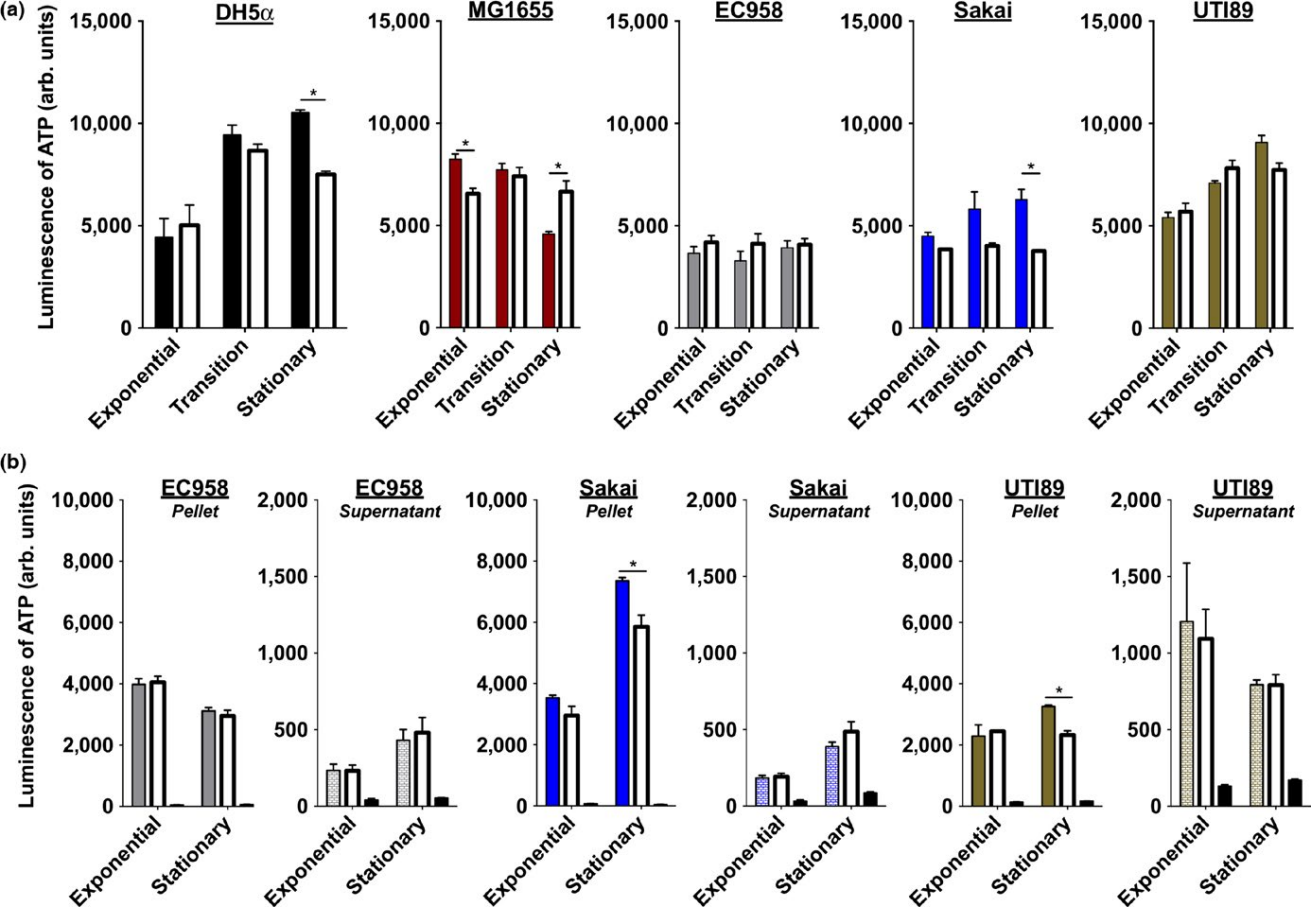
Blue light irradiation (BLI)_455_ is not completely bactericidal against *E. coli*. Viability assays, using an ATP release assay to measure ATP levels of unexposed and BLI_455_‐treated cells. ATP levels were measured using Promega's CellTiter‐Glo Kit to lyse cells and quantify ATP via luminescence. (a) The differences in relative ATP levels of unexposed (solid colored bars) and exposed (white bars) samples. (b) The differences in relative ATP in the pellet (intact) and supernatant (lysed) of unexposed (colored bars) and exposed (white bars) samples. The third bars are the controls, in which no bacteria was added, just the detection reagent and LB media (black bars). To determine if the differences in ATP levels were due to cell death or inhibition of replication. ATP in the supernatant and ATP in the pellet for both unexposed and exposed samples were measured. EC958, Sakai, and UTI89 were chosen as three representative strains to evaluate whether BLI is bacteriostatic or bactericidal because EC958 ATP levels were constant among the unexposed and exposed samples; Sakai and UTI89 had differences in levels of unexposed and exposed samples at each growth phase. Experiments were repeated three times. Error bars represent the standard error mean. Statistical analysis was performed using an unpaired, two‐tailed Student's *t* test. *, *p *<* *.05

First, the total ATP levels in bacterial samples were measured (Figure 3a). DH5α, which had the greatest overall reduction in CFU in response to BLI_455_ (Figure 2), exhibited no changes in the overall ATP levels during exponential and transition phase, but had approximately a 25% reduction in ATP levels at the stationary growth phase (Figure 3a). For MG1655, reduced ATP levels were observed in the exposed samples during the exponential growth phase and corresponded to the reduction in CFUs (Figure 2). The Sakai strain exhibited similar ATP levels between BLI and nonirradiated bacteria during exponential and transition phase and a modest reduction in ATP at stationary phase (Figure 3a). The ATP levels for strains UTI89 and EC958 were statistically insignificant from BLI_455_ (Figure 3a). These results suggested that BLI_455_ might induce altered ATP production or release in strains like DH5α, MG1655, and Sakai.

We next measured ATP released into the supernatant fraction (indicative of cell lysis), as well as ATP levels in cellular pellets for three representative strains: EC958, Sakai, and UTI89 (workflow is depicted in Fig. S4b). The ATP in the supernatant and cellular fractions was measured for both unexposed and exposed samples. EC958 was the least susceptible to BLI_455_ (Figure**** 2). Consistent with this observation and the insignificant changes in total ATP levels in response to irradiation (Figure 3a), we saw no significant changes in extracellular and intracellular levels of ATP between exposed and unexposed samples (Figure 3b). These observations validated that, for this particular *E. coli* strain, BLI_455_ is not effective at eliminating growth. For Sakai, there was no significant change in ATP levels during exponential phase between the unexposed and exposed supernatant fractions (Figure 3b), suggestive of no significant compromise to cellular membranes. Similarly, there were no significant changes in the ATP levels between the cellular fractions from exposed and unexposed cells from the exponential growth phase. This was in agreement with the modest reduction in CFUs observed during exposure in the exponential growth phase for strain Sakai (Figure 2). However, a significant reduction in intracellular ATP was observed for exposed fractions from the stationary growth phase, which was accompanied by a modest (but statistically insignificant) increase in the ATP levels in the corresponding supernatant fraction (Figure 3b). These data suggest that in the case of Sakai, BLI_455_ may exert some bactericidal effect (based on the modest increase in extracellular ATP), as well as bacteriostatic effects (based on the greater reduction in intracellular ATP that is not equivalent to the increase in extracellular ATP; note change in scale on the y‐axes between pellet and extracellular measurements). While strain UTI89 had greater reduction in CFUs compared to EC958 and Sakai in all growth phases (Figure 2), the differences in ATP of both intracellular and extracellular ATP mimicked the trends of Sakai (Figure 3b). There were no significant differences in ATP levels during exponential phase between the unexposed and exposed supernatant fractions (Figure 3b). As in Sakai, this suggests the cellular membranes are not significantly compromised. Similarly, in the exponential phase for the intracellular fraction, there were no significant changes in the ATP levels from exposed and unexposed cells (Figure 3b). Conversely, during the stationary phase, the ATP levels of the exposed cells were significantly lower than the levels in unexposed cells (Figure 3b). Combined with insignificant differences in the amount of lysed ATP in the supernatant, these findings suggest that BLI_455_ is bacteriostatic for UTI89. All together, the observed reductions in CFUs, with noncorresponding reductions in ATP levels suggest that BLI_455_ is either inhibiting replication of cells or drastically slowing the rate of replication. Slightly higher, but statistically insignificant, levels of ATP were observed in the supernatant of all tested strains in the stationary phase of growth (Figure 3b and S5). It is possible that some cell death is occurring, but the overall data suggest that the main mechanism of action is bacteriostatic and that the lower CFUs in exposed samples, as compared to unexposed samples, were not solely a result of cell death.

## 4 Discussion

In this study, we evaluated BLI_455_‐induced growth reduction and bactericidal activity during different growth stages of nonpathogenic, pathogenic, and multi‐drug‐resistant (extended spectrum beta‐lactamase producing) *E. coli* strains to determine intraspecies variation to a potential antimicrobial approach. We report that blue light‐mediated growth reduction of *E. coli* is minimal and also varies significantly as a function of strain background. Additionally, the bactericidal efficacy of BLI on all strains tested here demonstrated that BLI treatment at the wavelength and energy dose tested is mainly bacteriostatic. The same findings were observed with testing of BLI on preformed biofilms. The ability to inhibit outgrowth of preformed colony biofilms was also assessed by treating biofilms with BLI_455_ after 3 days of growth and measuring the biofilm diameters 48 hr after treatment. There were no differences in the size of the biofilms in unexposed and exposed samples of preformed biofilms with BLI_455_ with the same energy dose (120 J/cm^2^) used for treatment of planktonic cells (Fig. S7).

These findings suggest that BLI may be inducing a transient viable but nonculturable phenotype in a portion of the treated *E. coli* population and this effect leads to an apparent reduction in CFUs, but does not correspond to cell death. While BLI_455_ is used in numerous applications, including the treatment of jaundice in neonates, very few studies characterized the behavior of irradiated bacteria in a strain‐ and growth phase‐dependent manner. Numerous studies elegantly showed that there is extensive heterogeneity in bacterial populations, which changes as a function of external stimuli and growth phase (Anderl et al., 2003; Cabeen, 2014; Kolter et al., 1993; Mangan et al., 2006; Mouslim & Hughes, 2014; Navarro Llorens et al., 2010; Roop et al., 2003; Wang et al., 2014). In addition, bacteria that are commonly associated with humans and other vertebrate hosts are not typically growing exponentially, but are rather found in various growth phases, including extended stationary phase. Examining the bacterial responses in more physiologically relevant situations is essential, if we are to understand how to prevent or combat infections. The studies described in this work demonstrate that BLI treatment does not significantly impair *E. coli* viability and suggest that the use of BLI as an antimicrobial strategy may retard the growth of some strains, possibly including commensals, while conferring an advantage to pathogenic/opportunistic bacteria or bacteria found at a different phase of growth. For example, our findings demonstrated that UPEC strain EC958, a multi‐drug ST131 lineage *E. coli* (Totsika et al., 2011) and ETEC strain E9034A (Levine et al., 1984) were the least susceptible to the effects of BLI_455_; however, nonpathogenic E343 (Rúgeles et al., 2010) was also significantly resistant to the effects of BLI_455_. These findings have wide‐ranging ramifications in the way phototherapy may be used to treat or prevent infections.

The conventional model describing the BLI susceptibility mechanism is through damage from the generation of ROS, most notably singlet oxygen (Farr & Kogoma, 1991; Feuerstein et al., 2005, 2006; Liang et al., 2013; Lipovsky et al., 2009, 2010; Lubart et al., 2011; Malik et al., 1990; Marugán et al., 2010). Studies also indicate that low‐level stimulation of ROS can enhance proliferation of bacterial growth with the resulting daughter cells sometimes exhibiting altered replication rates (Dai et al., 2012; Lipovsky et al., 2009, 2010; Lubart, Lavi, Friedmann, & Rochkind, 2006; Lubart et al., 2011; Nussbaum et al., 2002). ROS generation could be a contributing factor in the reduced growth observed in response to BLI. Furthermore, lowering of the pH has been shown to protonate the cell membrane and “revive” bacteriostatic cells, suggesting that in a biological application and in the optimal environment, BLI‐treated bacteria may revert to a viable, replicating state (Darby et al., 2011; Hisert et al., 2004; Smith et al., 2005; Tian et al., 2005). Therefore, BLI_455_ treatment of *E. coli* (and possibly other gram‐negative bacteria) could lead to a viable but nonculturable state that can revive under appropriate conditions. This possibility raises questions with regard to long‐term consequences in cases when BLI is used on areas that teem with bacterial communities, such as the human skin. For example, BLI_455_ is routinely used to treat jaundiced neonates. What are the consequences of these treatments on the emerging skin microbiota? This question would be very interesting to pursue in a longitudinal fashion, especially in the context of prematurely born neonates and how BLI may transiently influence the skin microbiome.

In summary, our studies demonstrated a nonbactericidal effect of BLI on *E. coli* growth and demonstrated significant differences in intraspecies responses to BLI. Our studies also suggest that BLI induces a viable but nonculturable state in *E. coli* cells that may facilitate survival in the presence of BLI stress. However, transiently impairing the ability of *E. coli* to grow using BLI, may pose an attractive future means of preventing colonization on abiotic surfaces such as urinary catheters.

## Conflict of Interest

None declared.
