# Supplementary material for: Characterization of blue light irradiation effects on pathogenic and nonpathogenic Escherichia coli
Source: Microbiologyopen. 2017 Mar 22;6(4):e00466. doi: 10.1002/mbo3.466 (PMC5552948; doi:10.1002/mbo3.466)
Supplement: Supplementary file 1 [file MBO3-6-na-s001.pdf]

Supplementary Figures

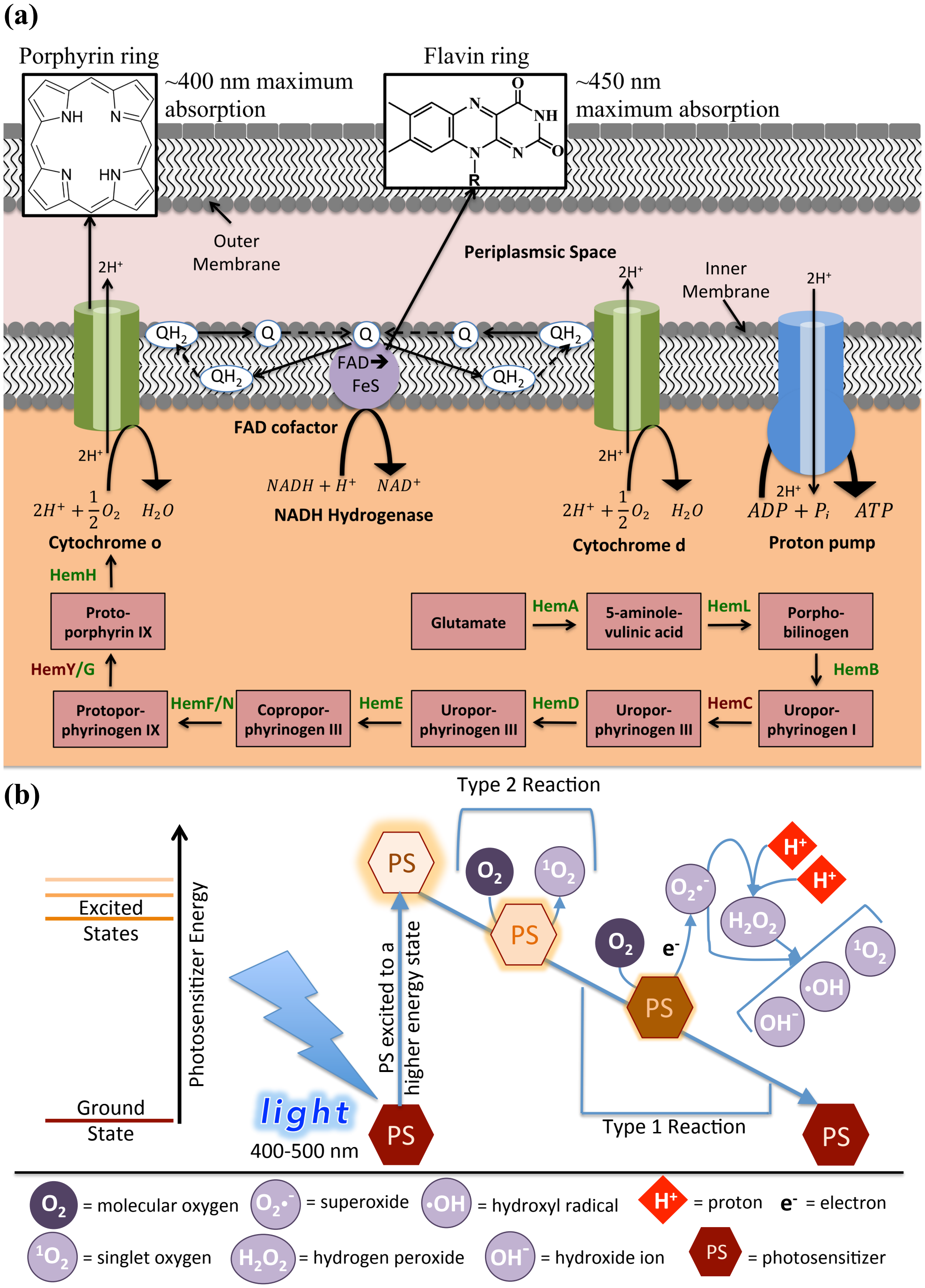

Figure S1. Photosensitizers play a major role in the response to visible light exposure. (a) Known endogenous photosensitizers (PSs) are found in the electron

transport chain (ETC) of bacteria. Porphyrins and flavins are two PSs, which absorb in the blue light wavelength range (400-500 nm). Energy is transported by ubiquinones (Q/QH_2). Protons (H^+) cross the membrane by reduction reactions and are actively transported by proton pumps. This proton gradient is the proton motive force (pmf) that triggers the ETC. Flavin-containing FAD and porphyrin-containing cytochromes are an active part of generating pmf. Hem enzymes are necessary for porphyrin production. The boxes in pink represent the biosynthetic pathway for the production of porphyrins and subsequently cytochromes. (b) Proposed mechanism of blue light-mediated bacterial reduction. Photosensitizers are excited upon light absorption and energy and/or electrons are released to form reactive oxygen species (ROS). A Type 2 reaction proceeds at high energy levels, while a multi-step Type 1 reaction occurs at lower energy levels and requires the presence of both protons and oxygen. The production of singlet oxygen ($^1O_2$) is thought to be the major player in the phototoxic response.

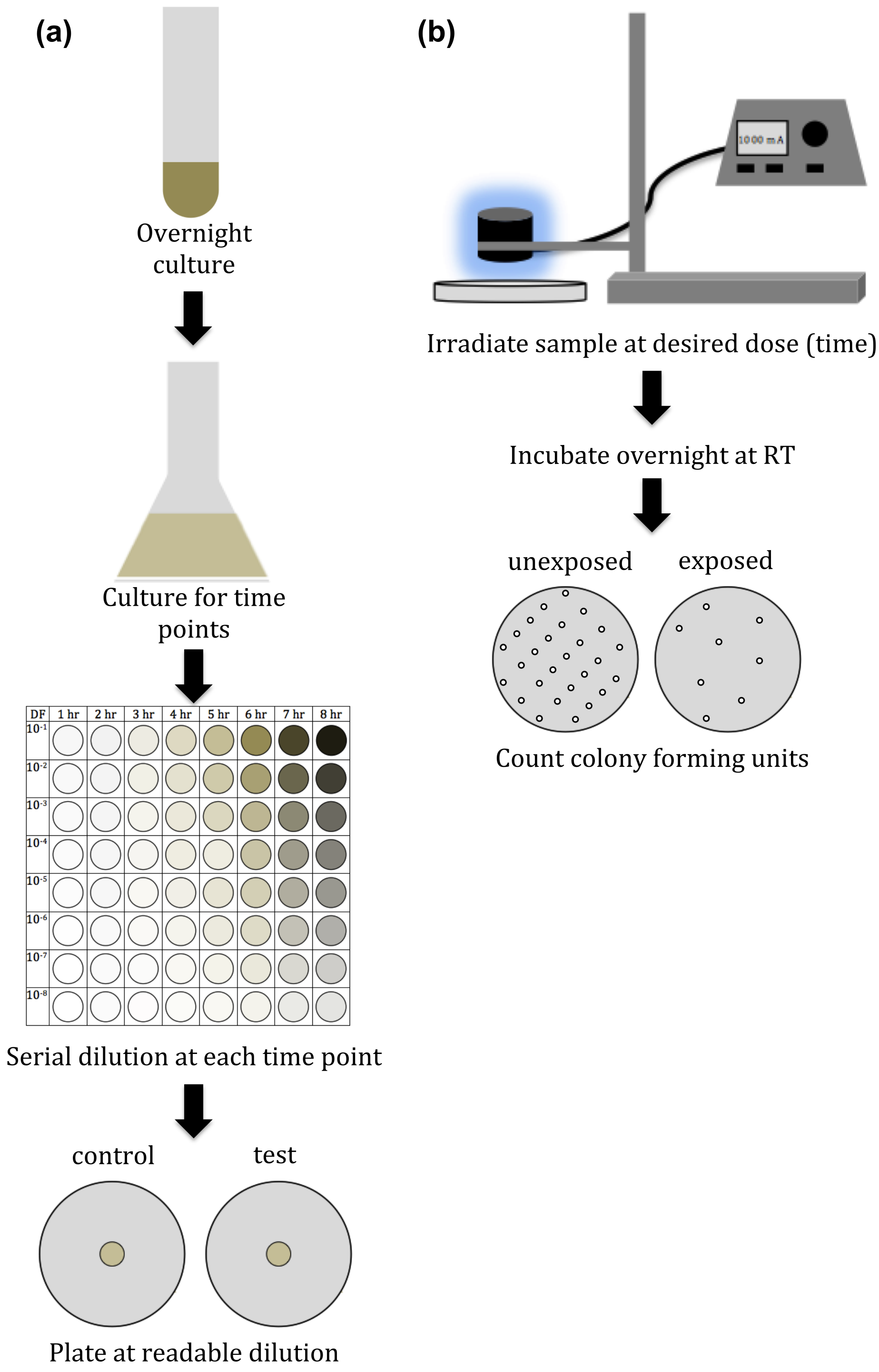

Figure S2. Experimental flow for blue light irradiation (BLI) experiments. a) Preparation procedure for BLI. b) BLI and quantitation of efficacy by comparing colony forming units of exposed vs. unexposed samples.

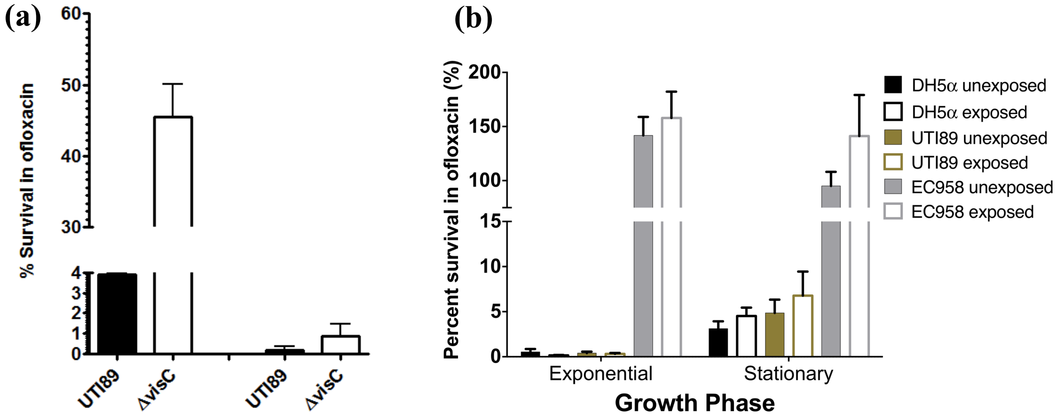

Figure S3. Evaluation of *E. coli* survival in ofloxacin with and without BLI. (a) Percent survival of ofloxacin after 5μg/mL treatment. UTI89Δ*visC* is known to produce a high percentage of persister cells. (b) Percent survival of a select group of lab-adapted (DH5α), uropathogenic (UTI89), and multi-drug-resistant (EC958) *E. coli* strains in ofloxacin (5 μg/mL) with and without exposure to BLI_{455}.

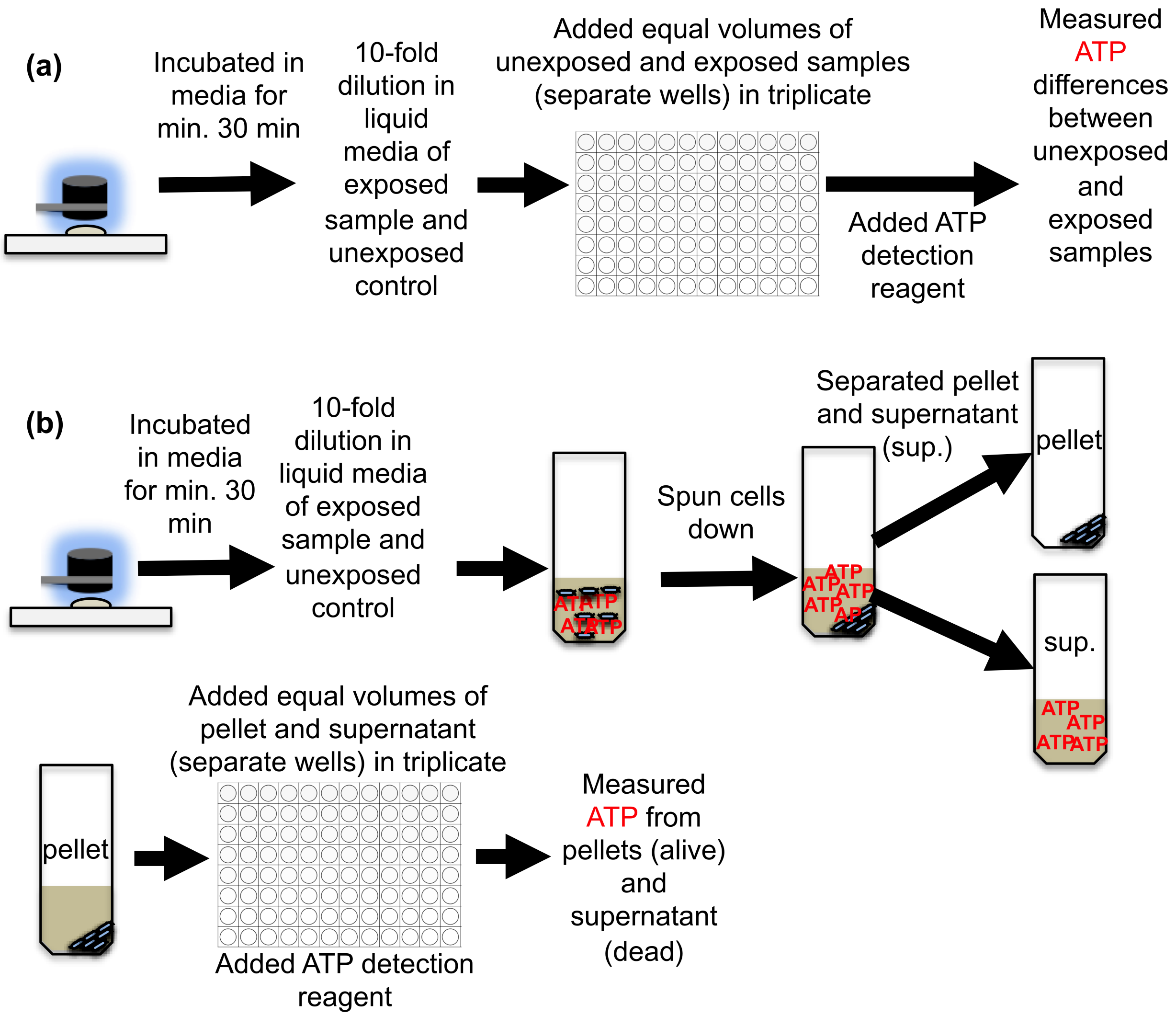

Figure S4. Workflow for ATP assay. (a) Relative ATP differences between unexposed and exposed samples were measured. (b) Relative ATP differences between unexposed and exposed supernatant and pellet samples were measured to determine whether total ATP differences were due to cell death or an inhibition in replication.

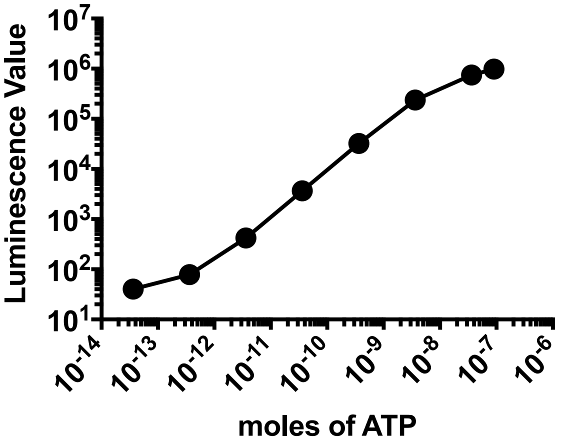

Figure S5. Standard curve for ATP measurements. A standard curve was developed using bio-grade ATP disodium salt hydrate (Sigma Aldrich). Known concentrations of ATP were added and the corresponding luminescence values were recorded.

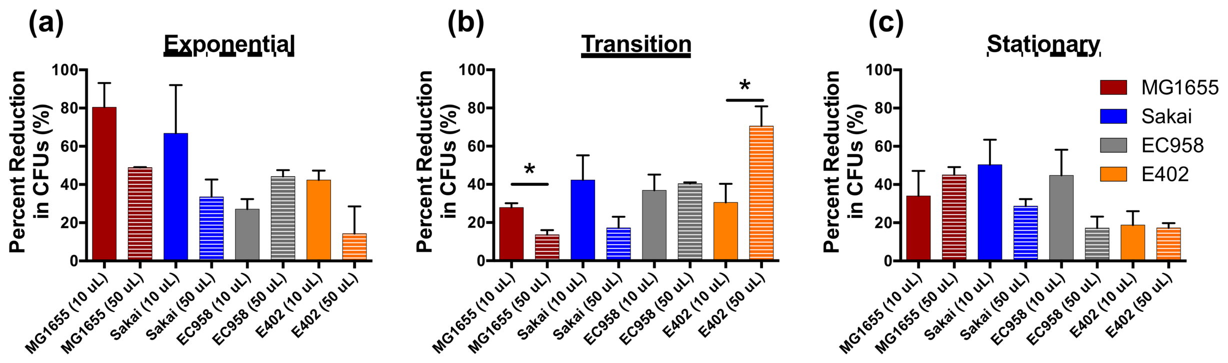

Figure S6. Percent reduction differences between CFUs on agar plate assay and glass slide assay. A few representative strains were chosen to show the minimal differences between BLI-induced reductions on 10 μL sample spots used in the agar plate assay and 50 μL glass slide assay. (a) Exponential, (b) Transition, and (c) Stationary growth phases. All experiments were repeated 3 times and analyzed via an unpaired, two-tailed Student's *t*-test. *, $P < 0.05$.

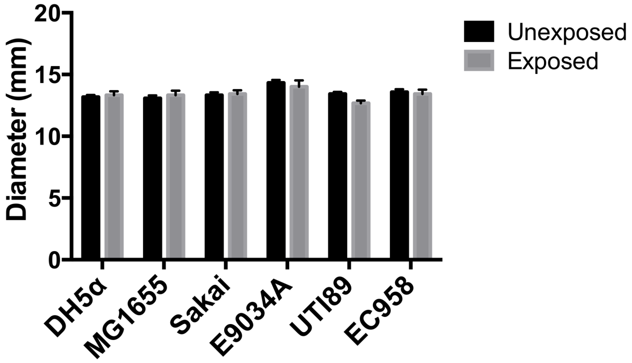

Figure S7. Effects of BLI on preventing pre-formed biofilms. BLI at 455 nm, 120 J/cm^2 was applied to colony biofilms after 3 days of growth. Biofilm diameters were measured 2 days after BLI (5 days after starting growth). There were no statistically significant differences in in biofilm diameter for the unexposed and exposed biofilms.
